# Clinical and translational implications of immunotherapy in sarcomas

**DOI:** 10.3389/fimmu.2024.1378398

**Published:** 2024-06-25

**Authors:** Federica Recine, Silvia Vanni, Alberto Bongiovanni, Valentina Fausti, Laura Mercatali, Giacomo Miserocchi, Chiara Liverani, Federica Pieri, Roberto Casadei, Davide Cavaliere, Pina Tiziana Falbo, Danila Diano, Toni Ibrahim, Alessandro De Vita

**Affiliations:** ^1^ Medical Oncology Unit, Azienda Ospedaliera “San Giovanni Addolorata”, Roma, Italy; ^2^ Preclinic and Osteoncology Unit, Biosciences Laboratory, IRCCS Istituto Romagnolo per lo Studio dei Tumori (IRST) “Dino Amadori”, Meldola, Italy; ^3^ Clinical and Experimental Oncology, Immunotherapy, Rare Cancers and Biological Resource Center, IRCCS Istituto Romagnolo per lo Studio dei Tumori (IRST) “Dino Amadori”, Meldola, Italy; ^4^ Osteoncology, Bone and Soft Tissue Sarcomas and Innovative Therapies Unit, IRCCS Istituto Ortopedico Rizzoli, Bologna, Italy; ^5^ Pathology Unit, “Morgagni-Pierantoni” Hospital, Forlì, Italy; ^6^ Orthopedic Unit, “Morgagni-Pierantoni” Hospital, Forlì, Italy; ^7^ General and Oncologic Surgery, “Morgagni-Pierantoni” Hospital, Forlì, Italy; ^8^ Radiology Unit, IRCCS Istituto Romagnolo per lo Studio dei Tumori (IRST) “Dino Amadori”, Meldola, Italy

**Keywords:** tumor immune microenvironment, immunotherapy, immune-based classification, tumor-related genes, immune cells, tumor infiltrating lymphocytes

## Abstract

Immunotherapy has emerged as promising treatment in sarcomas, but the high variability in terms of histology, clinical behavior and response to treatments determines a particular challenge for its role in these neoplasms. Tumor immune microenvironment (TiME) of sarcomas reflects the heterogeneity of these tumors originating from mesenchymal cells and encompassing more than 100 histologies. Advances in the understanding of the complexity of TiME have led to an improvement of the immunotherapeutic responsiveness in sarcomas, that at first showed disappointing results. The proposed immune-classification of sarcomas based on the interaction between immune cell populations and tumor cells showed to have a prognostic and potential predictive role for immunotherapies. Several studies have explored the clinical impact of immune therapies in the management of these histotypes leading to controversial results. The presence of Tumor Infiltrating Lymphocytes (TIL) seems to correlate with an improvement in the survival of patients and with a higher responsiveness to immunotherapy. In this context, it is important to consider that also immune-related genes (IRGs) have been demonstrated to have a key role in tumorigenesis and in the building of tumor immune microenvironment. The IRGs landscape in soft tissue and bone sarcomas is characterized by the connection between several tumor-related genes that can assume a potential prognostic and predictive therapeutic role. In this paper, we reviewed the state of art of the principal immune strategies in the management of sarcomas including their clinical and translational relevance.

## 1 Introduction

Sarcomas represent a heterogeneous group of cancers, encompassing more than 100 histological subtypes (1, 2). Recently, many more entities have been identified due to the discovery of new molecular profiling. Due to the complexity of their clinical presentation, the optimal management of sarcomas must be referred in dedicated Centers with a multidisciplinary approach (3).

The majority of localized sarcomas can be treated with radical surgery, in selected cases also in combination with (neo) adjuvant chemotherapy and radiation treatments. The cornerstone of treatment in the metastatic setting is anthracycline based-chemotherapy or targeted therapies according to specific histotype, with a median overall survival of 12–18 months (4).

Even though immunotherapy is one of the most promising treatments in Oncology, the effectiveness of the novel immune strategies in the management of sarcomas is still under debate and the oncological outcomes remain poor in the advanced stages of disease.

Indeed, the clinical application of immunotherapy led to controversial results, reflecting the complexity of the Tumor Immune Microenvironment (TiME) among the different multiple histotypes in sarcomas. Therefore, understanding the mechanisms underlying the response/resistance to immunotherapy and the biological characterization of TiME is critical to improve the clinical management of these neoplasms.

The aim of this paper is an overview of the principal strategies targeting the TiME as potential therapeutic approach in the management of sarcomas and starting point for thorough biological knowledge in these neoplasms.

## 2 The clinical (and translational) relevance of tumor immune microenvironment in sarcomas

Tumor immune microenvironment (TiME) plays a key role in cancer progression and treatment response of tumors (5–7).

TiME is characterized by complex interplays between different elements including cancer cell subpopulations, immune infiltrate, vascular, and also extracellular matrix components.

Indeed, it has been shown that the extracellular matrix can have a key role in the tumorigenesis, metastatic process and resistance to treatments (8).

On the other hand, tumor cells can gradually model the TiME to escape immune surveillance through multiple mechanisms including, among others, alterations in the antigen presentation system, enhancing the negative immune regulatory pathways and recruiting tumor-promoting immune cells.

The induced tumor cell immunosuppression is crucial to block host immunity, and alterations in the balance between the immune components and pro- and antitumor inflammatory mediators may determine tumor progression. The pro-tumor immune cells play an important role in blocking antitumor immune responses and shaping an immunosuppressive microenvironment and are represented mainly by the regulatory T cells (Tregs), myeloid-derived suppressor cells (MDSCs), tumor-associated macrophages (TAM), in particular M2-polarized macrophages, and group 2 innate lymphoid cells (ILC2s)., the majority of antitumor immune cells include effector T cells, such as cytotoxic CD8 + T cells and effector CD4 + T cells, natural killer cells (NK), dendritic cells (DC), and M1-polarized TAM (9).

Despite great heterogeneity across different cancer types and populations, the central role of the TiME in tumor pathogenesis and proliferation is quite similar.

The tumor immunogenicity of sarcomas has been observed firstly in the 1890s by Dr. William Coley, who noted tumor regression in some sarcoma patients as a consequence of bacterial infections (10). Tumor sarcoma heterogeneity has a profound correlation with the TiME, with a relevant clinical impact and a potential therapeutic role. Sarcoma TiME seems to be frequently infiltrated by a high variability of immune cell populations, including Tumor-associated macrophages (TAMs), cytotoxic T lymphocytes (TLS) and cancer-associated fibroblasts (CAFs). TAMs represent the major components in the tumor sarcoma microenvironment and can influence tumor growth and progression as well as cancer-related inflammation. TAMs can be present in the TiME as precursors including blood monocytes, monocyte-related myeloid-derived suppressor cells (M-MDSCs), and tissue-resident macrophages that are activated and differentiated into TAMs in response to cancer-related inflammatory factors (11).

Several preclinical experiences showed that TAMs exhibit two phenotypes, M1 and M2-like, correlated to cancer prognosis, growth and tumor progression. The M1-phenotype displays anti-tumorigenic function, whereas the M2-like profile is correlated to pro-tumorigenic phenotype. Indeed, the clinical relevance of TAMs is particularly evident in bone as well as soft tissue sarcomas (12, 13).

Among bone sarcomas, osteosarcoma is the most common histotype and commonly arises in adolescents and young adults. It shows a variety of histological subtypes, including conventional (osteoblastic, chondroblastic, and fibroblastic types), telangiectatic, small cell, low-grade central, parosteal, periosteal, high-grade surface, and secondary osteosarcoma. Several studies demonstrated that the dysregulation of M1/M2 in favor of M1-phenotype TAMs is associated with localized osteosarcoma and a M2 profile is associated with a worse prognosis in patients with this neoplasm (14–17).

In a similar way, preclinical evidences suggested that the high infiltration of M2 TAMs determines a worse outcome in STS patients, including Undifferentiated Pleomorphic Sarcoma (UPS), Synovial Sarcoma (SS) and Leiomyosarcoma (18–21).

The relevance of intratumoral macrophages and in general of inflammatory system in modulating tumor aggressiveness and response to drug treatments in soft tissue sarcomas is highlighted also by the prognostic value of systemic inflammatory indices, such as neutrophil-to-lymphocyte ratio (NLR), platelet-to-lymphocyte ratio (PLR), lymphocyte-to-monocyte ratio (LMR). Indeed, in a recent study involving 99 STS patients who received second-line treatment after progressing to anthracycline, it has been shown that LMR is a specific predictor of Trabectedin efficacy, suggesting its potential use in daily clinical practice. Moreover, a possible correlation between LMR levels and the percentage of intratumoral macrophages was reported as well (22).

It has become clear that the tumor-infiltrating immune population but also immune-cell-related genes in TiME of sarcomas may influence the prognosis and responses to immunotherapy (23).

CAFs represent another cell population which can be part of the sarcoma tumor microenvironment that is implicated in the blockade of T-cell infiltration. CAFs in sarcomas have been underinvestigated due to challenges in distinguishing CAFs from malignant cells. Current evidences have recognized the potential role of CAFs in the tumor microenvironment by hindering T-cell infiltration and creating an immunosuppressive tumor microenvironment (24).

The improved availability of genomic technologies has led to a better knowledge of sarcoma biology. According to a genomic point of view, sarcomas can be classified in two groups: 1) sarcomas with a single driver molecular alteration, defined as sarcomas with “simple genetics” and 2) sarcomas with a complex genomic profile, or with “complex genetics”. The first group includes sarcomas that are characterized by specific driver molecular alterations, mainly oncogenic gene fusions, but also activating or inactivating mutations, or gene amplifications. Some of these examples are sarcomas that present chromosomal translocations leading to fusion oncogenes, including myxoid liposarcoma with the fusion oncoprotein FUS/EWSR1-DDIT3 (SSX-SS18), alveolar rhabdomyosarcoma, GIST (e.g., PAX3/7- FOXO1 and KIT mutations respectively). The second group comprises large numbers of chromosomal and point mutations, reflecting high genomic instability. These events can influence the immunogenicity of sarcomas and the related response to immunotherapy. Within this group there are for example pleomorphic/spindle cell morphologies including pleomorphic sarcoma, DDLPS, LMS and pleomorphic leiomyosarcoma (PLPS), pleomorphic rhabdomyosarcoma, malignant peripheral nerve sheath tumor (MPNST), myxofibrosarcoma (MFS) and UPS (25). In general, the genomic instability in sarcomas may lead to an inflammatory signaling with pleiotropic effects, including a potential increase of sensitivity of cancer cells to immune checkpoint inhibitors. For instance UPS, which is one of the most frequent sarcomas of the trunk and extremities with a high potential of metastasization, displays a complex genomic profile and an immune “hot” microenvironment. Indeed, clinical trials have demonstrated tumor response in UPS patients treated with ICI-immunotherapy (26, 27).

Even though the whole landscape of sarcoma microenvironment phenotypes remains unknown, several studies have analyzed sarcoma gene expressions to characterize the immune infiltrate, their clinical relevance and the correlation between tumor genetic and immune signatures of sarcomas (28, 29). The Cancer Genome Atlas (TCGA) Research Network has comprehensively performed an integrated genomic analysis of soft tissue sarcomas, showing several alterations in copy number of genes, methylation, RNA, and protein in these neoplasms. This large-scale analysis provided relevant information about genes involved in immune response and inflammation in specific histotypes. Among others, well-differentiated (ALT/WDLPS) and dedifferentiated liposarcoma (DDLPS) were associated with the amplification of MDM2 and CDK4, leiomyosarcoma (LMS) correlated to smooth muscle differentiation, undifferentiated pleomorphic sarcoma (UPS) was characterized by the lack of any differentiation line and myxofibrosarcoma (MFS) showed fibroblastic differentiation with myxoid stroma. In this analysis an immune signature was identified in each sarcoma type to improve the understanding regarding the potential implications of novel therapeutic targets, such as immunotherapies. Furthermore, the authors determined an immune infiltration score for various immune cells based on their gene expression signatures. UPS, MFS and DDPLS had the highest median macrophage scores among the other types, while DDLPS had the highest CD8 score, and LMS of soft tissue (STLMS) as described in the paper had the highest PD-L1 score. These immune-gene signature differences are correlated to the various sensitivity to immunotherapy and patients survival (30). Several evidences have pointed out that the expression of high levels of CD8+ lymphocytes and PD-L1 are correlated to the sensitivity of ICIs as described better in the next paragraph (31).

## 3 Histology-driven therapeutic approach: is there a role for immunotherapy in sarcomas?

The majority of sarcomas is characterized by a non-inflamed microenvironment and is considered immunologically “cold”. With the recent introduction in oncology of cancer immunotherapy, interest has been focused also on the sarcoma field (27).

Immunotherapy, including checkpoint inhibitors and adoptive cellular therapies, has had promising activity in a selected group of sarcomas. Among the novel immune therapies, Immune Checkpoint Inhibitors (ICIs) have shown apparently limited activity in sarcomas, however the patient selection is crucial to improve clinical outcomes of these drugs. Several clinical trials have been conducted in a wide variety of sarcoma histotypes, revealing controversial results.

The PEMBROSARC study, a clinical multicohort single arm phase II study, evaluating the role of anti PDL1, Pembrolizumab, associated with metronomic cyclophosphamide in patients with soft tissue sarcoma (STS), have led to disappointed results in terms of 6-month non progression rate (NPR), objective response rate (ORR), progression free survival (PFS), overall survival (OS). The subsequent analysis, evaluating patients selected on the presence of B cells in the immune compartments called tertiary lymphoid structures (TLS), reported an improvement of the results in all of the previous clinical outcomes (32, 33). This evidence confirmed significant clinical activity of ICIs in STS which are characterized by the presence of the TLS. TLS represent a crucial site for tumor immunogenicity in sarcomas. Some studies support the influence of TLS in the promotion of T and B cells and their central role in shaping the immune microenvironment of tumors (34–36).

In the non-randomized multicenter multicohort phase II SARC028 study 80 patients, 40 with soft tissue sarcoma and 40 bone sarcoma, were treated with Pembrolizumab in monotherapy, showing an objective response in one among 22 patients with osteosarcoma, and one among 5 chondrosarcoma patient on a total of 40 bone sarcoma patients (5%). None of the 13 Ewing’s sarcoma patients showed an objective response.

A clinical response in 18% of patients among the STS patients cohort was observed. One patient with histotype of undifferentiated pleomorphic sarcoma experienced complete response. To further investigate the different responses to immunotherapy, tumor biopsies before and during immunotherapy were performed in patients with STS. The results revealed higher density of tumor-infiltrating T cells and TAMs expressing PD-L1 in patients that responded to pembrolizumab compared to not responders. In the expanded cohort of the SARC028 trial, the majority of the responders were affected by UPS and DDLPS, emphasizing the correlation of favorable immune profile and the activity of pembrolizumab.

The previous transcriptomic analysis including data from >600 STSs identified the sarcoma histotypes characterized by high expression of a B cell-related gene signature and the presence of tertiary lymphoid structures, that were classified as “immune high” microenvironment tumors. The proposed sarcoma immune classification pointed out that the presence of B cells in the TLS could be potential biomarkers associated with a favorable survival and also response to immunotherapy in patients with sarcomas (30).

Moreover, it has been reported that the presence of Tumor infiltrating lymphocytes (TILs) in the TiME can positively predict outcomes in sarcoma patients. TILs can be correlated to PDL1 expression in 12–65% of sarcomas, considered in patients with the presence of B cell infiltration in the tumor even though limited sample size studies are available in this regard and further studies are needed (37, 38).

The single-arm phase II AcSè Pembrolizumab study have been demonstrated that the activity of the immunotherapeutic drug is improved in selected rare and ultra-rare sarcoma histotypes, including chordoma and alveolar soft-part (ASPS), rhabdoid tumors, SMARCA4 deficient sarcomas and desmoplastic small round cell tumors (DSRCT). The results of the trial showed an activity of the drug in these specific sarcoma histotypes and the confirmation that the PD-1/PD-L1 pathway can be a potential predictive therapeutic target in this population (39, 40).

Nonetheless, the role of PDL1 expression as a marker of response to immunotherapy currently remains controversial considering the inter and intra-variability of this potential biomarker in each sarcoma histotype.

Some trials have tested combination therapies with the aim to escape resistance mechanisms at various levels of the cascade. A phase II trial evaluated the combination of pembrolizumab and axitinib, a VGFR inhibitor, with the majority of responses in patients with ASPS. Tumor biopsies from ASPS patients revealed the presence of high infiltration of TIL and PD-L1 expression, suggesting an “immune high” profile correlated to the favorable activity of the immunotherapy in this population (41). Preclinical data suggested that trabectedin has synergistic effects with PD1 inhibition and may influence the tumor microenvironment, enhancing the activity of immune-modulating agents and reducing tumor-associated macrophages (42, 43). The NITRASARC study investigated the safety and efficacy of the combination between trabectedin and the anti-PD 1 agent nivolumab in patients with advanced STS, with a group of L-sarcomas patients. The safety was manageable and clinical outcomes were improved with the combination, even though the observed activity in this study was not superior compared to the expected outcomes with trabectedin alone (44). The combination of nivolumab with the doxorubicin/dacarbazine chemotherapy regimen in leiomyosarcoma patients was explored by M. Broto et al. based on the evidence that doxorubicin can have the potential to induce immunogenic cell death and synergize with PD1 pathway. This trial revealed that nine patients out of 36 had responses with the combination, showing also a manageable safety profile (45).

Other studies evaluated the association between immunotherapy and antiangiogenic therapy, for example the Alliance A091902 study, a phase 2 study exploring the combination of nivolumab, with cabozantinib, a multikinase inhibitor, in pretreated taxane-based chemotherapy patients with advanced angiosarcomas. The hypothesis of the study is that cabozantinib may act with the PD-1 expression in regulatory T cells, modulating the TiME to a mostly “responsive” immune phenotype when combined with nivolumab. Treatments in the experimental arm were well tolerated with the objective response rate of 59% (46). Cabozantinib was also studied with ipilimumab and nivolumab compared to the antiangiogenic drug alone. The results suggested a potential additive effect of the combination with nivolumab and ipilimumab (47). Other sarcoma histotypes in which ICI-based immunotherapy has a relevant role, seem to be alveolar soft part sarcoma (ASPS), UPS and Kaposi sarcoma. A recent meta-analysis showed that high response rates to immunotherapy were observed in ASPS and in UPS with an ORR of 0.35 and 0.20, respectively as well as Kaposi sarcoma. Moreover, the efficacy of ICI in ASPS has been demonstrated in several studies that confirmed the favorable clinical outcomes with ICIs in patients with this ultra-rare sarcoma (48).

These results underline that both STS carrying complex karyotypes and STS related to specific fusion transcripts can be susceptible to immunotherapy (38). In this regard, translocation-driven sarcomas, epigenetic regulation of the transcriptional program might manipulate the tumor immunity to improve responses to ICI therapy (23).

The evidence that a limited number of sarcoma patients in each histological sarcoma subtype experiences a clinical benefit from ICI treatment may be correlated to the genetic and immunological heterogeneity that dominate each single histology. Overall, these data underline the importance to deeply investigate the molecular features linked to immune evasion to select the candidate tumors for immunotherapy also within the same histotype. Moreover, this meta-analysis pointed out that early association of ICIs with chemotherapy or with TKI shows improved ORR rates in sarcoma patients, further stressing the value of the combination of ICIs with drugs characterized by immunomodulating properties. On the basis of results obtained in other tumors, clearly indicating that non-immune pathways could impact response to ICI therapy, an additional combination therapy that can be proposed for STS is the use of drugs targeting CDKs as CDK4/6 with palbociclib (46). In fact, pathways related with CDK activation are crucial for sarcomagenesis, but they also have an active role in limiting immune surveillance in human tumors and likely in sarcomas, as well. Moreover, it was demonstrated that CDK4/6 pathway is correlated to a potential resistance mechanism to anti-PD1 therapy in melanoma (48) and increased activity of the CDK4/6 inhibitor CDKN2A correlates with response to PD-L1 blockage in RCC and NSCLC patients (46).

We reported in **Table 1** the selected clinical trials evaluating immunotherapy as single agent or in combination.

**Table 1:** Selected Clinical Trials with ICIs monotherapy or combination in sarcomas.

Clinical Trial	Phase	Agent/Intervention	Histotypes	Results/outcomes (weeks/months)
**Italiano et al. Amended PEMBROSARC**	Phase II	pembrolizumab with metronomic cyclophosphamide	35 TLS positive STS (12 WDLPS/DDLPS, 4 LMS, 6 UPS, 3 EpS, 10 other histotypes)	PFS: 6 ms
**Blay et al. French AcSé 2021** (38)	Phase II	pembrolizumab	98 rare STS (34 chordoma, 14 ASPS, 11 SMRT, 8 DSCRT, 31 other histotypes)	PFS 2.75 ms OS 19.7 ms
**Tawbi et al.** **SARC028**	Phase II	pembrolizumab	40 STS cohort 40 BS cohort	PFS: OS: 18 weeks 49weeks 8 weeks 52weeks
**D’Angelo et al.** **Alliance A091401**	Phase II	nivolumab/ipilimumab vs. nivolumab	Nivolumab/ipilimumab: 42 sarcomas (3 AS, 4 BS, 14 LMS, 2 LPS, 6 SCS, 2 SS, 6 UPS/MFH, 1 unspecified sarcoma, 4 others) Nivolumab: 43 sarcomas (5 BS, 15 LMS, 3 LPS, 2 unspecified sarcoma, 5 SCS, 2 SS, 5 UPS, 6 others)	PFS: OS 1.7 ms 10.7ms
**Martin-Broto et al. IMMUNOSARC 2022**	Phase I/II	nivolumab + sunitinib	52 STS (9 SS, 8 UPS, 7 clear cell sarcoma, 7 SFT, 7 EpS, 5 AS, 4 ESMCS, 4 ASPS, 1 EHET)	PFS: 5.6 ms
**Palmerini et al. IMMUNOSARC 2020**	Phase II	nivolumab + sunitinib	40 (BS 17 OS, 14 CS, 8 ES, 1 bone UPS, 4 DDCS)	PFS: OS 3.7 ms 14.2ms
**Wilky et al., 2019** (39)	Phase II	Pembrolizumab + axitinib	33 STS (12 ASPS, 6 LMS (4 uterine), 5 High-grade PS, 2 DDLPS, 8 other histotypes)	PFS: 4.7 ms OS: 18.7 ms
**Andreou et al. NITRA-SARC 2023**	Phase II	nivolumab + trabectedin	Group A—43 STS (28 LMS and 15 LPS) Group B—49 (12 UPS, 11 SCS, 6 FMS, 5 SS, 4 EpS)	PFS: 5.5 ms OS 18.7 ms PFS 2.3 ms OS 5.6 ms

OS, Overall survival; PFS, Progression-free survival; STS, soft tissue sarcoma; BS, bone sarcoma.

It is important to note that in these studies presented at ASCO, patients were not selected by any biomarkers or immune-markers, such as TIL or TLS. In general, it is desirable that in the future the sarcoma patients clinical trials would be stratified and selected by (immune) biomarkers in order to improve clinical outcomes.

## 4 Molecular signatures of sarcomas: prognostic and predictive role of the immune-related genes

One common mechanism in cancer is the down-regulation of cell surface expression of MHC class I over time as an immune escape strategy thanks to which they mitigate the immune response directed against tumor cells, therefore greatly influencing the outcome of T cell-mediated immunotherapy (49). Indeed, the suppression of the expression of MHC-I is known to prevent both the antitumor effect of type II IFN response and the infiltration of CD8+ T cell, enabling tumor cells to evade recognition and destruction by cytotoxic T lymphocytes (50, 51).

This has been reported also for several sarcomas. Indeed, Berghuis et al. observed a substantial down-regulation of MHC expression in the majority of Ewing Sarcoma cell lines as well as in 79% of EWS tumor samples analyzed - including metastatic tissues highlighting a potential explanation for the low response rates to immunotherapy reported in STS (52). A very recent study showed the STC2-mediated down-regulation of MHC-I molecules in osteosarcoma as a mechanism to achieve immune evasion by suppressing type II interferon response and reducing CD8+ T cells infiltration (53). In addition, a reduction of both HLA-A and HLA-B genes was reported also in UPS, MFS, LMS, Undifferentiated sarcoma and MLPS compared to respective healthy tissue (54). Interestingly, the down-regulation of class I MHC mediated antigen processing in MFS and UPS patients has been recently correlated with anthracycline resistance of patient-derived primary cultures (55). Moreover, also in Kaposi sarcoma the inhibition of immune surface molecules such as MHC-I is one of the multiple strategies utilized by Kaposi sarcoma-associated herpesvirus to evade the human immune system (56). Of note, it has been recently reported that CDK4/6 inhibitors can revert this mechanism in gammaherpesvirus-infected cell lines, providing the rational for the potential application of these drugs in Kaposi sarcoma as well (57).

In recent years, many efforts have been made in an attempt to better characterize immune-related genes profile of STS in order to predict immunotherapy efficacy.

Indeed, T cells can be modified *ex vivo* to express cancer-antigen specific T cell receptors (TCRs), generating TCR-engineered T cells. Since TCRs are HLA-restricted, only antigens presented by a specific HLA molecule can be recognized. Therefore, the efficacy of TCR-T cell therapy as adoptive cellular therapy can be influenced by the patient’s HLA type. On the other hand, chimeric antigen receptors (CARs) recognition of surface proteins is mediated by an antibody-derived scFv domain. The binding then leads to T cell activation as well as additional stimulation of conjugated costimulatory receptors (58). Therefore, in CAR-T cells is the antigen recognition and cytotoxic stimulus are independent on MHC presentation.

In soft tissue sarcomas, the use of T cells recognizing New York esophageal squamous cell carcinoma 1 specific antigen, presented by HLA A02* (NY-ESO-1), was shown to be safe and effective in metastatic synovial sarcoma and myxoid liposarcoma (59). In this regard, very recently Rosenbaum et al. investigated the prognostic and predictive role of selected HLA-A*02 genotypes in 23 patients with metastatic synovial sarcoma (SS). They observed that SS patients with an HLA-A*02 genotype had a shorter OS when survival was analyzed from the time of metastasis, indicating this genotype as a negative prognostic factor and that HLA genotype is a relevant predictive biomarker for SS patients interested in receiving genetically engineered T cells (60).

Moreover, in a previously cited work, Mosca et al. identified a shared haplotype of rare HLA class I allelic variants in a cohort of 40 soft tissue sarcoma patients (54). They observed that HLA-A24*:10-B73*:01 haplotype was the most common in the population, while HLA-B mRNA and HLA-A were down-regulated or deleted, suggesting that STS select rare alleles of HLA-I loci and/or delete HLA-B locus. This finding could help increase the effectiveness of immunotherapy in sarcomas. Indeed, it has been recently reported that selected tumor epitopes can be used to prime T cells to recognize specific peptide-HLA complex, thereby selectively targeting tumor cells. In particular, common tumor haplotypes could be exploited as targets for immunotherapeutic approaches such as bispecific T-cell engagers (BITEs). These molecules are made of two single-chain variable fragments, one binding to a T-cell-specific molecule, whereas the other binds to a tumor-associated antigen. Therefore, BITEs are able to improve the patient’s immune response to tumors by retargeting T cells to tumor cells (61).

A different approach was followed by Gu and colleagues. In a recent work, they built an immune gene-related prognostic model for STS using gene expression data from the Cancer Genome Atlas and GEO datasets based on five immune-related prognostic genes (IFIH1, CTSG, STC2, SECTM1, and BIRC5) (62). Based on this model, the authors performed risk stratification for 573 STS patients and predicted their responsiveness to immunotherapy. In particular, they demonstrated that low-risk patients with higher overall survival time had higher expression of immune-stimulating molecules, higher stimulating cytokines and corresponding receptors, higher innate immunity molecules, and stronger antigen-presenting capacity. On the other side, high-risk patients had a high tumor mutation burden, which did not significantly influence survival.

Interestingly, several studies focused on the role of immune-related genes in osteosarcoma (OS). Indeed, dysregulation of immune cell infiltration in the TiME is known to contribute to the progression of OS, including metastasis and drug resistance (63). A recent study focused on the construction of a risk model able to predict the prognosis and pilot therapeutic strategies for OS, taking advantage of TARGET and GEO databases to analyze the expression of genes related to immune cell infiltration (64). The analyses identified 225 differentially expressed genes (DEGs), 175 upregulated and 50 downregulated, in the tissues with a high infiltration of resting dendritic cells versus low-infiltration groups in OS specimens. Moreover, a protein-protein interaction network analysis was performed and 94 genes were identified as resting dendritic cell-associated genes. Among these, four genes (AOC3, CDK6, COL22A1, and RNASE6) constructed a resting dendritic cell signature able to predict OS prognosis and therapy guidance. In a similar work, aimed to screen the metabolic features associated with prognoses, a hexosamine biosynthesis pathway-related gene signature (GPI, PGM3, UAP1, OGT and MGEA5) was identified. The signature resulted correlated with immune infiltration and prognosis prediction in OS patients (65), and thus could help clinicians in patients stratification and drive the future development of immunotherapy and targeted therapy in osteosarcoma.

## 5 Conclusions

Tumor cells reside in a complex environment of other multiple cells, including immune cells such as macrophages and CAFs, infiltrating immune cells, and blood vessels, which contribute to the cancer growth and to the mechanisms of treatment resistance. TiME can have a potential therapeutic role in the management of tumors, including sarcomas. Therefore, targeting the TiME may be crucial for reducing tumor growth and enhancing therapeutic responses. Moreover, understanding the immune adaptive changes in the TiME during tumor development and the crosstalk between tumor cells and immune microenvironment can help to shed light on targeted therapeutic strategies and improve response rates of treatments, including immunotherapy. Sarcomas TiME is composed of numerous and heterogeneous immune cells, including macrophages and TIL (5–9).

Immunotherapy has deeply revolutionized the management of different solid and hematological tumors including among all, lung, renal, melanoma and urothelial tumors.

Sarcomas are characterized by high heterogeneity in terms of histological subtypes, prognostic factors and clinical behavior, together with a relative low frequency of these solid malignancies. These differences are consequently reflected on the immunological features of the histotypes exposed to immunotherapies, which also harbor a variety of genetic aberrations affecting the TME composition. For the above reasons a clear interpretation of the role of immunotherapy in the treatment of sarcoma is challenging and it is not already elucidated.

The clinical benefit of immunotherapy in the management of these tumors showed limited results. In this regard the observed results are often controversial and for the majority the intrinsic resistance to ICI is correlated to a “cold” immune landscape in the TiME sarcomas.

The combination of immunotherapy and other therapeutic agents, including chemotherapy or antiangiogenic therapy, has been reported able to convert a “cold” into a “hot” TiME in sarcomas (23, 33, 38). Another mechanism which can improve the ICI responsiveness is the presence of B cells in the TIL, which has been correlated to an improvement of the results in the clinical oncological outcomes in sarcoma patients treated with ICI. In particular, patients likely to respond to immunotherapy exhibited higher expression of PD-L1 receptors in T cells and in the tumor associated macrophages (38, 46). However, the majority of clinical trials, also the latest ones, did not include a proper patient selection, potentially leading to disappointing results.

It is advisable in the future to perform a better stratification of the patients with sarcomas to improve clinical outcomes in these heterogeneous neoplasms.

Overall, the key to better understand the biology of a heterogeneous group as sarcomas and to ameliorate their clinical outcome, is to focus on a translational approach leading to an appropriate selection of patients.

In conclusion, the role of immunotherapy in sarcoma treatment is still controversial and further studies are needed to better investigate its potential benefit for selected cases. Despite some important advancements have been achieved in recent years, deeper analyses focusing on specific histotypes need to be carried out in order to fully explore the potentiality of immunotherapy.

## Author contributions

FR: Conceptualization, Writing – original draft, Writing – review & editing. SV: Writing – original draft. AB: Data curation, Writing – review & editing. VF: Data curation, Writing – review & editing. LM: Writing – original draft, Writing – review & editing. GM: Writing – original draft. CL: Data curation, Writing – review & editing. FP: Data curation, Writing – review & editing. RC: Data curation, Writing – review & editing. DC: Data curation, Writing – review & editing. PF: Data curation, Writing – review & editing. DD: Data curation, Writing – review & editing. TI: Data curation, Writing – review & editing. AD: Conceptualization, Writing – original draft, Writing – review & editing.

## Glossary

**Table:**

ASPS	alveolar soft tissue sarcoma
CAF	cancer-associated fibroblast
CAR-T	chimeric antigen receptor-T cell
DC	dendritic cell
DDLPS	dedifferentiated liposarcoma
DSRCT	desmoplastic small round cell tumor
ICI	immune checkpoint inhibitor
ILC2s	2 innate lymphoid cells
IRG	immune-related gene
LMS	leiomyosarcoma
MDSC	myeloid-derived suppressor cell
MFS	myxofibrosarcoma
MLPS	myxoid liposarcoma
M-MDSC	monocyte-related myeloid-derived suppressor cell
MPNST	peripheral nerve sheath tumor
NK	natural killer cell
NPR	non progression rate
ORR	objective response rate
OS	overall survival
PDL1	programmed death ligand 1
PFS	progression free survival
PLPS	pleomorphic leiomiosarcoma
SS	synovial sarcoma
STS	soft tissue sarcoma
TAM	tumor-associated macrophages
TCR	T cell receptor
TiME	tumor immune microenvironment
TLS	tumor infiltrating lymphocytes
Treg	regulatory T cell
UPS	undifferentiated pleomorphic sarcoma
